# Supplementary material for: Causal Relationship between Aging and Anorexia Nervosa: A White-Matter-Microstructure-Mediated Mendelian Randomization Analysis
Source: Biomedicines. 2024 Aug 16;12(8):1874. doi: 10.3390/biomedicines12081874 (PMC11351342; doi:10.3390/biomedicines12081874)
Supplement: Supplementary file 1 [file biomedicines-12-01874-s001.zip › Supplementary Figure/Figure S4/Singe SNP plot of MR analysis for TL to ubm-b-2080.pdf]

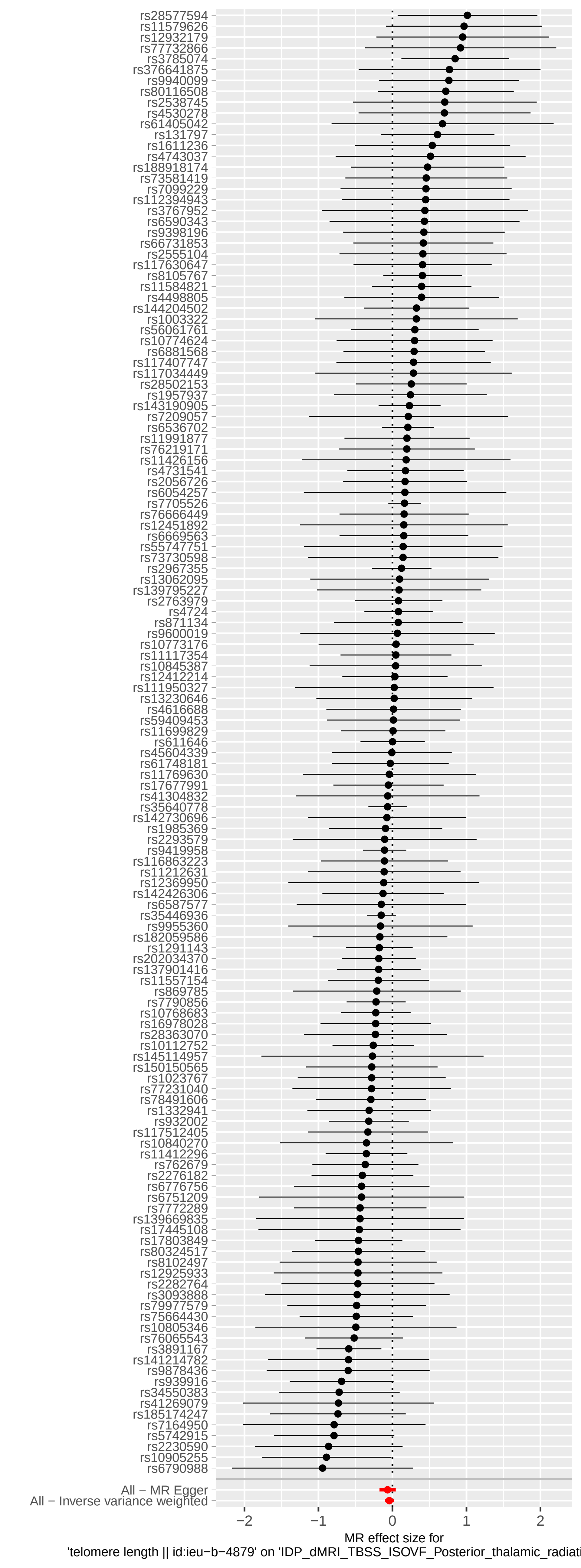
rs28577594
rs11579626
rs12932179
rs77732866
rs3785074
rs376641875
rs9940099
rs80116508
rs2538745
rs4530278
rs61405042
rs131797
rs1611236
rs4743037
rs188918174
rs73581419
rs7099229
rs112394943
rs3767952
rs6590343
rs9398196
rs66731853
rs2555104
rs117630647
rs8105767
rs11584821
rs4498805
rs144204502
rs1003322
rs56061761
rs10774624
rs6881568
rs117407747
rs117034449
rs28502153
rs1957937
rs143190905
rs7209057
rs6536702
rs11991877
rs76219171
rs11426156
rs4731541
rs2056726
rs6054257
rs7705526
rs76666449
rs12451892
rs6669563
rs55747751
rs73730598
rs2967355
rs13062095
rs139795227
rs2763979
rs4724
rs871134
rs9600019
rs10773176
rs11117354
rs10845387
rs12412214
rs111950327
rs13230646
rs4616688
rs59409453
rs11699829
rs611646
rs45604339
rs61748181
rs11769630
rs17677991
rs41304832
rs35640778
rs142730696
rs1985369
rs2293579
rs9419958
rs116863223
rs11212631
rs12369950
rs142426306
rs6587577
rs35446936
rs9955360
rs182059586
rs1291143
rs202034370
rs137901416
rs11557154
rs869785
rs7790856
rs10768683
rs16978028
rs28363070
rs10112752
rs145114957
rs150150565
rs1023767
rs77231040
rs78491606
rs1332941
rs932002
rs117512405
rs10840270
rs11412296
rs762679
rs2276182
rs6776756
rs6751209
rs7772289
rs139669835
rs17445108
rs17803849
rs80324517
rs8102497
rs12925933
rs2282764
rs3093888
rs79977579
rs75664430
rs10805346
rs76065543
rs3891167
rs141214782
rs9878436
rs939916
rs34550383
rs41269079
rs185174247
rs7164950
rs5742915
rs2230590
rs10905255
rs6790988
All − MR Egger
All − Inverse variance weighted
−2
−1
0
1
2
MR effect size for
'telomere length || id:ieu−b−4879' on 'IDP_dMRI_TBSS_ISOVF_Posterior_thalamic_radiati
